# The Effect of Long-Term Storage on the Physiochemical and Bactericidal Properties of Electrochemically Activated Solutions

**DOI:** 10.3390/ijms14010457

**Published:** 2012-12-24

**Authors:** Gareth Robinson, Robin Thorn, Darren Reynolds

**Affiliations:** Centre for Research in Biosciences, Department of Applied Sciences, University of the West of England, Bristol BS16 1QY, UK; E-Mails: gareth2.robinson@uwe.ac.uk (G.R.); robin2.thorn@uwe.ac.uk (R.T.)

**Keywords:** ECAS, long term storage, physiochemical, bactericidal

## Abstract

Electrochemically activated solutions (ECAS) are generated by electrolysis of NaCl solutions, and demonstrate broad spectrum antimicrobial activity and high environmental compatibility. The biocidal efficacy of ECAS at the point of production is widely reported in the literature, as are its credentials as a “green biocide.” Acidic ECAS are considered most effective as biocides at the point of production and ill suited for extended storage. Acidic ECAS samples were stored at 4 °C and 20 °C in glass and polystyrene containers for 398 days, and tested for free chlorine, pH, ORP and bactericidal activity throughout. ORP and free chlorine (mg/L) in stored ECAS declined over time, declining at the fastest rate when stored at 20 °C in polystyrene and at the slowest rate when stored at 4 °C in glass. Bactericidal efficacy was also affected by storage and ECAS failed to produce a 5 log_10_ reduction on five occasions when stored at 20 °C. pH remained stable throughout the storage period. This study represents the longest storage evaluation of the physiochemical parameters and bactericidal efficacy of acidic ECAS within the published literature and reveals that acidic ECAS retain useful bactericidal activity for in excess of 12 months, widening potential applications.

## 1. Introduction

Concerns regarding the spread of antimicrobial resistance amongst microorganisms (particularly within healthcare and agricultural environments), coupled with limitations associated with existing biocides continues to drive the development of novel disinfectants. Electrochemically activated solutions (ECAS), also referred to as electrolyzed oxidizing (EO) water [1], mixed oxidant (MIOX) solutions [2] and electrochemically activated (ECA) water [3] are generated from the electrolysis of NaCl solution in an electrochemical cell. Solutions originating from reactions that occur at the anode have proven microbicidal efficacy resulting from the formation of HOCl, which is considered to be the primary antimicrobial agent. Such solutions typically exhibit redox potentials (ORP) between +800 mV and +1200 mV, and pH values in the range of 2 to 5 [4].

ECAS have a broad spectrum of activity. They have been shown to be highly effective against healthcare associated bacterial pathogens including methicillin resistant *Staphylococcus aureus*, *Pseudomonas aeruginosa* [5] and vancomycin resistant *Enterococcus faecalis* [6]. They have also demonstrated rapid activity against bacterial endospores including those of *Clostridium difficile* and *Bacillus atrophaeus* [5], *Bacillus cereus* [7] and *Bacillus anthracis* [8]. In addition to bacterial and sporicidal activity, ECAS have been shown to be active against human norovirus [9], hepatitis B virus [10] and HIV [11]. Fungicidal activity against *Aspergillus flavus* [12], *Candida albicans* [13] and a range of plant pathogenic fungi [14] have been demonstrated in addition to efficacy against *Cryptosporidium parvum* oocysts [2] and staphylococcal enterotoxin A [15]. The active antimicrobial components of ECAS have been reported to include HOCl, hydroxyl radical, and other short-lived oxidative moieties and comparisons have been made with the respiratory burst from phagocytic cells of the mammalian immune system. These have been reported to cause bacterial membrane degradation, and inactivation of cellular protein, lipids and nucleic acid [4].

ECAS are reported to be safe for the human body and the environment [16], degrading to salt and water during chemical relaxation and are effectively inactivated by organic matter [4]. ECAS have been evaluated extensively within the food industry, both in process decontamination and tested on food itself and are widely considered to be environmentally friendly [17,18], fulfilling the role of a “green biocide” through reduced free chlorine use [19] and a reduction in the requirement for disposal of toxic chemicals [20]. Furthermore, the basic material requirements for the production of ECAS, namely NaCl, water and electricity means that it can be produced on demand, obviating the need for transport and storage of large volumes of liquid disinfectant and the associated environmental implications associated with this. Whilst production on demand can be considered one of the advantages of ECAS, there may be situations where transport and storage are necessary, for example where no electricity, salt or water supply is easily available, or where it is not cost effective to have a generator onsite. An understanding of the impact of storage of these solutions is required in order that all potential applications of these novel biocides can be fully explored. Due to their HOCl content, ECAS can be categorised with other chlorine releasing agents, such as sodium hypochlorite. Sodium hypochlorite is an important high level chlorine releasing disinfectant, though there are long-standing safety concerns regarding the formation of disinfection by products such as trihalomethanes following reaction with organic matter [21]. The production of chlorine releasing agents can generate chemical by products such as sodium hydroxide, or chloramines (from the reaction of ammonia with aqueous chlorine), which are toxic to aquatic life. Furthermore, the use of the chlor alkali process using mercury cells to produce sodium hypochlorite is a cause for concern as these cells have been shown to release mercury into the environment [22]. The ECAS generation technology used in this study does not use mercury. Furthermore, ECAS has been shown to have greater efficacy at lower free chlorine concentrations than sodium hypochlorite [23] mitigating some of the environmental concerns around chlorine releasing agents since ECAS can be utilised at a lower free chlorine concentration.

Due to the broad spectrum of activity, safety and environmental compatibility, suggested healthcare applications for ECAS include treatment of periodontal disease [24], wound irrigation [25], instrument disinfection [6] and environmental decontamination [4]. Within the food industry, applications include disinfection of food processing equipment [26] and direct washing of fruits and vegetables [27], fish [28], poultry [29] and meat [30]. Furthermore, ECAS have been shown to have useful biofilm removal properties [31]. Disadvantages to ECAS as general purpose disinfectants include concerns about potential corrosiveness [32], substantially reduced efficacy in the presence of organic soiling [5,33,34] and declining bactericidal activity if not used shortly after production [35,36].

A number of studies have investigated the physiochemical properties of ECAS under short term storage in the absence of antimicrobial efficacy testing. A study by Len *et al.* [36] investigated the chlorine loss, redox potential (ORP) and pH of ECAS when stored in glass jars, open or closed, agitated or non agitated, in light or dark conditions. For open containers, the physiochemical parameters were measured periodically over 4 days and for closed containers over 58 days. Hsu and Kao [37] studied pH, ORP, total residual chlorine, dissolved O_2_, conductivity, and Na^+^ and Cl^−^ concentrations of ECAS stored in glass bottles, for 12 days (opened periodically) or 21 days (opened only for measurements on first and last day). Another study investigated hypochlorous acid loss from ECAS stored in Erlenmeyer flasks at a range of temperatures from 25 °C to 40 °C for 22 days [38].

There have been a limited number of studies that have investigated the antimicrobial efficacy of solutions stored in glass bottles for periods of up to 30 days. Fabrizio and Cutter [30] tested the pH, ORP, free and total chlorine of ECAS stored in glass bottles at 4 °C or 25 °C over 3 days. Bactericidal efficacy was also tested, but only at a single time point 24 h post-production. The pH, ORP, conductivity, available chlorine, dissolved oxygen and bactericidal efficacy of ECAS stored in glass bottles under open or closed and light or dark conditions was tested over a 30 day period by Cui *et al.* [39], with bactericidal efficacy tested on the first and last day of the study. Nisola *et al.* [35] measured pH, ORP and free chlorine of ECAS stored in dark glass bottles (open or closed) at 25 °C over 30 days relating the physiochemical parameters to the bactericidal efficacy which was tested using fresh solutions only. The current study is concerned with characterising the changes in pH, ORP, free and total available chlorine and basic bactericidal activity of ECAS stored over a period of 13 months. Basic bactericidal activity was assessed using EN 1040:2005 “Chemical disinfectants and antiseptics—Quantitative suspension test for the evaluation of basic bactericidal activity of chemical disinfectants and antiseptics—Test method and requirements (phase 1)” representing a European standard method for establishing whether a chemical disinfectant or antiseptic does or does not have a basic bactericidal activity. ECAS were stored at two temperatures (4 °C and 20 °C) and in different materials (glass and polystyrene). This represents the first study in the literature where ECAS have been stored for an extended duration (in excess of 12 months), in ambient and cold conditions, in both glass and plastic containers and where bactericidal efficacy and the physiochemical parameters have been measured contemporaneously throughout.

## 2. Results and Discussion

### 2.1. Redox Potential (ORP)

**Figure 1:**
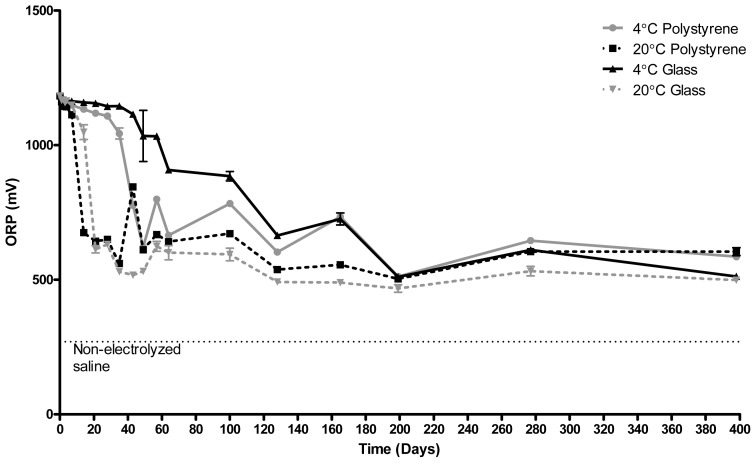
Redox potentials (ORP) of electrochemically activated solutions (ECAS) (mV) stored in polystyrene or glass containers at 20 °C or 4 °C for 398 days. *N* = 3 ± SD.

During long term storage, the ORP of ECAS is reduced substantially from that at the point of production, though the rate of reduction depends upon storage conditions, as shown in Figure 1. Irrespective of storage material, ECAS stored at 20 °C demonstrated a greater reduction in ORP over a shorter time period than that stored at 4 °C. The ORP of ECAS stored in polystyrene dropped at a slightly faster rate than that stored in glass. After 14 days, the ORP of ECAS stored in polystyrene and glass at 4 °C was 675 mV and 1048 mV respectively, compared to 1133 mV and 1159 mV respectively for polystyrene and glass at 4 °C. ECAS stored in glass at 4 °C showed the slowest decline in ORP retaining an ORP of greater than 1000 mV for 57 days compared to that of 35 days for ECAS stored in plastic at 4 °C, 14 days for ECAS stored in glass at 20 °C and 7 days for ECAS stored in polystyrene at 20 °C. It is also clear that, although in the short term, storage material and temperature greatly influence the retention of a high ORP, following long term storage the redox potential of the stored solutions begins to equilibrate.

### 2.2. pH

**Figure 2:**
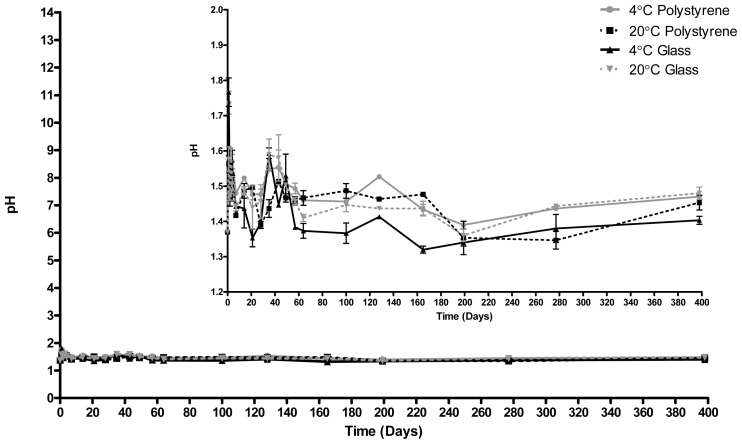
pH of ECAS stored in polystyrene or glass containers at 20 °C or 4 °C for 398 days. *N* = 3 ± SD.

Figure 2 shows the pH of ECAS under long term storage with the inset graph displaying the same data on a limited pH scale of 1.2 to 2.0. Despite the decline in ORP over the same time period, the pH of ECAS remained stable over 398 days, irrespective of storage conditions.

### 2.3. Free Chlorine

**Figure 3:**
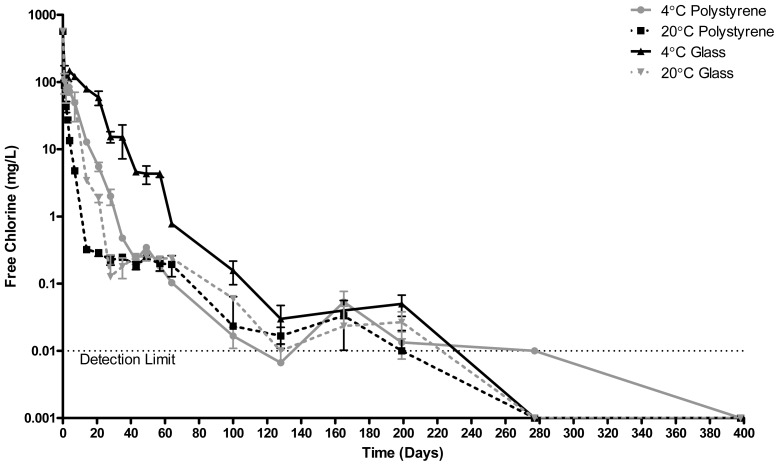
Free chlorine concentration of ECAS (mg/L) stored in polystyrene or glass containers at 20 °C or 4 °C for 398 days. *N* = 3 ± SD.

Figure 3 shows the free chlorine level of stored ECAS over a 398 day period. In all cases, the greatest loss in free chlorine occurred over the first 24 h. As was the case with ORP, the rate of loss is the slowest when stored in glass at 4 °C. The detection limit of the test procedure is 0.01 mg/L. By 277 days, all samples except for those stored in polystyrene at 4 °C had free chlorine levels of <0.01 mg/L. ECAS stored in polystyrene at 4 °C had 0.01 mg/L of measurable free chlorine remaining. The time point at which free chlorine levels within the stored ECAS became not statistically different from zero varied according to storage material and temperature. For polystyrene at 20 °C, this was day 7, for glass at 20 °C day 14, for polystyrene at 4 °C day 21 and for glass at 4 °C day 28.

### 2.4. Antibacterial Activity

**Figure 4:**
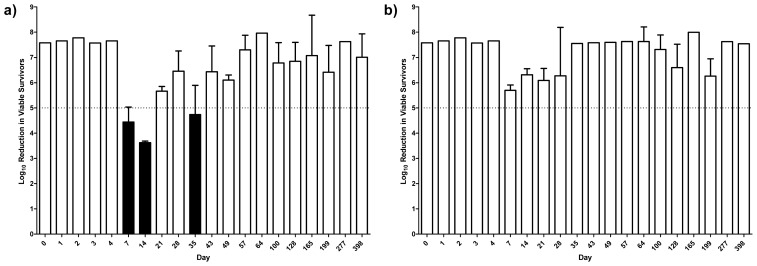

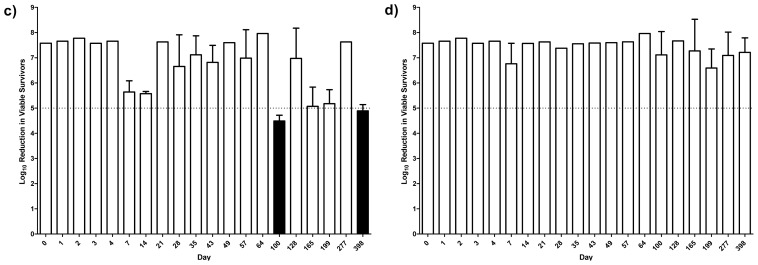
Log_10_ reduction in viable *P. aeruginosa* ATCC 9027 when treated with ECAS stored in polystyrene containers at 20 °C (**a**) or 4 °C (**b**) or in glass containers at 20 °C (**c**) or 4 °C (**d**) for 398 days. *N* = 3 ± SD. Broken line indicates 5 log_10_ reduction required to meet EN1040:2005. Black bars show instances where 5 log_10_ reduction was not achieved.

Figure 4 shows the log_10_ reduction in viable bacteria when using stored ECAS against *P. aeruginosa* ATCC 9027. Over the first 4 days of storage, zero counts were recorded on all recovery plates indicating that the population of surviving bacteria within the test was fewer than 10 cfu/mL. Basic bactericidal activity is retained in all storage conditions, though the consistency of the result varies depending on storage material and temperature. When stored in polystyrene at 20 °C (Figure 4a), ECAS failed to produce a log_10_ reduction of 5 (the definition of basic bactericidal activity under EN 1040:2005) on three occasions, compared to two occasions when stored in glass at 20 °C (Figure 4c). When stored at 4 °C in polystyrene or glass (Figure 4b,d respectively), a 5 log_10_ reduction was achieved at every time point. There was greater variability in the log_10_ reduction achieved when stored at 20 °C than at 4 °C and the log_10_ reduction was consistently higher when stored in glass compared to polystyrene. Over the 398 day storage period, the mean log_10_ reduction achieved under each storage condition (± SD) was 6.634 ± 1.204 in polystyrene at 20 °C, 6.720 ± 1.135 in glass at 20 °C, 7.196 ± 0.6947 in polystyrene at 4 °C and 7.441 ± 0.3402 in glass at 4 °C.

### 2.5. Discussion

The physiochemical properties of ECAS depend upon the characteristics of the electrochemical cell from which it is produced and its operating parameters [4]. Electrolysis of NaCl in these electrochemical cells yields a solution from both the anodic and cathodic chamber of the cell. Acidic ECAS are produced from the anode and can yield solutions with an ORP of greater than +1100 mV and a pH of lower than 2.7. Whereas the cathodic solutions are basic with a pH of 11 or more and an ORP lower than −800 mV and weak antimicrobial activity compared to acidic ECAS [40]. A modified variant of acidic ECAS can also be produced by directing some of the anodic output back into the cathode chamber, producing a neutralised anolyte often referred to as neutral electrolyzed water [39]. The antimicrobial properties of acidic ECAS have been widely reported, though one of the primary concerns regarding these solutions is the rapid loss of antimicrobial activity post-production [16]; however, this lack of residual activity is likely to contribute to the environmental compatibility of ECAS. Neutralised acidic ECAS have shown greater antimicrobial activity during shelf life trials than their acidic counterparts [41]. Superior antimicrobial activity during storage can be attributed to the greater stability of neutral ECAS, as these solutions generally have lower ORP values at the point of production than those of acidic ECAS, which is considered to be the primary contributor to their bactericidal activity [42].

The data generated from this study suggest that that both temperature and storage material significantly influence the rate at which ORP and chlorine decline in acidic ECAS, with temperature having a greater influence on long term storage than the storage material. Fabrizio and Cutter [30] also determined that ECAS were more stable when stored at 4 °C than when stored at 25 °C over three days, with ORP remaining stable over this time period and chlorine levels increasing in the stored solution. The ECAS used in their study had a pH of 2.6, an ORP of 1150 mV and a free chlorine concentration of 50 ppm (at point of production) and they found that freshly produced and “aged” EO water (stored for 24 h at 4 °C) both demonstrated potent bactericidal activity. As with our study, pH remained constant throughout short term storage. A study by Len *et al.* [36] showed that ECAS (EO water) stored at 25 °C lost 40% of its “active chlorine” after 58 days storage under closed conditions. However, in our study, by this time point, in excess of 99.9% of free chlorine had been lost under all storage conditions compared to levels recorded at the point of production. Len *et al.* stored their ECAS in 1.7 L volumes in glass vessels compared to the 30 mL samples stored in our study. The ECAS used in the current study had an ORP of 1180 mV, a pH of 1.37 and a free chlorine concentration of 570 mg/L at the point of production compared to that of 1020–1120 mV, pH 2.5–2.6 and 53–56 mg/L chlorine used by Len *et al.* They suggested that the primary mechanism of chlorine loss under closed conditions (in the absence of evaporation) was self-decomposition of chlorine species in solution. The ECAS samples used in the study by Len *et al.* were pH adjusted up to 9 though they found that more rapid chlorine loss was observed at lower pH values. This could account for the rapid chlorine loss observed in the current study. This study found that pH remained stable throughout storage. Len *et al.* observed only slight reductions in ORP, as did Hsu and Kao [37] who also observed a stable pH during storage, but more rapid loss of chlorine in those ECAS with the lowest pH and highest chlorine levels at the point of production.

Cui *et al.* [39] studied neutral and acidic ECAS stored for 30 days, also finding that pH remained stable during the storage period. The acidic ECAS used in their study had a pH of 3.01, ORP of 1079.1 and chlorine concentration of 25 mg/L and they found that in addition to pH, ORP remained stable over the storage period under closed conditions, whilst chlorine concentrations decreased by 80% and 96% in dark and light conditions respectively and to undetectable levels in open conditions after 30 days. Evaluating bactericidal activity against *Salmonella* Enteritidis and *Escherichia coli* O157:H7 demonstrated that acidic ECAS stored for 30 days was no less effective than at the point of production. Although the authors recognised that acidic ECAS have potent bactericidal activity due to lower pH and higher ORP than neutral ECAS, they concluded that available chlorine may be the primary factor responsible for bactericidal activity. This is in contrast to the work of Venkitanarayanan *et al.* [43] who suggested that ORP, pH and chlorine act in combination. Our study also demonstrated bactericidal activity in the absence of detectable chlorine, supporting the assertion that the bactericidal activity of these solutions is not wholly dependent on chlorine concentration. Nisola *et al.* [35] stored neutral and acidic ECAS for 30 days at 25 °C and tested them against *E. coli* O157:H7 and *Salmonella* Typhi. The acidic ECAS was produced with a pH of 2.0, ORP of 1271 mV and chlorine concentration of 173 mg/L. At the point of production acidic ECAS was the superior biocide, the authors suggesting that the low pH sensitised bacterial membranes to chlorine penetration. Under closed conditions a slight decrease in ORP was observed with a concomitant increase in pH and chlorine, which stabilised after 10 days. Following 30 days storage, 1.1 and 1.0 log_10_ reductions in *S. typhi* and *E. coli* were observed respectively compared to >8 log_10_ reductions at the point of production. Conversely, in the current study a >7 log_10_ reduction in viable *P. aeruginosa* was measured after 398 days storage in all conditions with the exception of polystyrene at 20 °C. The ECAS evaluated in the current study failed to achieve a >5 log_10_ reduction in viable bacteria on five occasions, all of which occurred when stored at 20 °C though on four of those occasions solutions stored under the same conditions achieved a >5 log_10_ reduction at a later date. ORP and free chlorine were found to decline more slowly at 4 °C than at 20 °C and also more slowly when stored in glass vessels than polystyrene vessels. Failure to achieve a >5 log_10_ reduction on these independent test dates may have been due to headspace being left in the vessels, although every effort was made to exclude this at the point of production. The presence of headspace would have allowed more rapid volatilisation of Cl_2_ from solution compared to the suggested self-decomposition in the absence of airspace [36]. The ECAS used in the current study are capable of meeting or exceeding a modified EN 1040:2005 up to 277 days post production under all storage conditions tested and up to 398 days in three of the four conditions tested. This activity is most reliably achieved with samples stored at 4 °C rather than 20 °C, and in glass rather than polystyrene vessels and whilst storage in glass vessels would be impractical on a day-to-day basis, this research has also demonstrated retention of basic bactericidal activity when stored in plastic for a period of up to 13 months. Under the storage conditions tested, temperature had more of an influence on the physiochemical parameters and antimicrobial efficacy of ECAS than storage material. Previous work has shown that acidic ECAS are potent bactericidal and sporicidal agents [5]. Whilst high-level efficacy may be dependent on a combination of ORP, pH and chlorine concentration [43], the physiochemical parameters of solutions stored under differing conditions equilibrate after long term storage, suggesting that for long-term storage applications, storage temperature and material may be less important than for short-term storage applications.

## 3. Experimental Section

### 3.1. Solutions and Storage Conditions

ECAS was produced from a saturated sodium chloride solution and softened mains water in an electrochemical cell (Bridge Systems Ltd.: Fife, UK) at a rate of 200 mL/min from the anodic chamber and 400 mL/min from the cathodic chamber. ECAS was produced at an ORP of 1183.1 mV, a pH of 1.37 and a free chlorine concentration of 570 mg/L. ECAS was collected in two sets of containers; 30 mL thick-walled glass Universal bottles with rubber-sealed aluminium screw caps, and 30 mL polystyrene Universal bottles with high density polyethylene screw caps. The containers were filled sufficient to exclude air, sealed and stored under dark conditions at either 4 °C or 20 °C. At the every time point, three samples of ECAS stored under each of the four conditions were analysed for ORP, pH, free and total chlorine and tested for bactericidal activity.

### 3.2. Physiochemical Properties

The redox potential and pH of ECAS were measured immediately after collection (Sartorius PT-10P portable meter with PYP12 pH electrode and CEPTRL/87 ORP electrode, calibrated according to manufacturer’s instructions; Sartorius: Epsom, UK). At each time point, three ECAS samples stored in glass and plastic at 4 °C and 20 °C were tested similarly. Furthermore, free and total available chlorine were assayed using a chlorine DPD test (Palintest: Gateshead, UK) following appropriate dilution in sterile deionized water.

### 3.3. Bactericidal Activity

At each time point, three ECAS samples stored in glass and plastic at 4 °C and 20 °C were subjected to a modified version of EN 1040:2005, *Staphylococcus aureus* ATCC 6538 was removed from the regimen as previous research had shown it to be more susceptible to the bactericidal activity of ECAS than *P. aeruginosa* ATCC 9027 (data not shown). A dilution-neutralization assay was undertaken in accordance with EN 1040:2005. A stock culture of *P. aeruginosa* ATCC 9027 was streaked onto Tryptone Soya Agar (TSA; Oxoid: Basingstoke, UK) and incubated at 37 °C for 18–24 h. A single, isolated colony was subcultured onto TSA for 18–24 h. Colonies were suspended in diluent (1 g/L Tryptone, 8.5 g/L NaCl) to a density of 1.5–5 × 10^8^ cfu/mL (test suspension). 1 mL of test suspension and 1 mL water were added to a Universal bottle and incubated at 20 °C for 2 min ± 1 s. 8 mL of stored ECAS (equilibrated to 20 °C) was added for a contact time of 5 min ± 10 s followed by immediate removal to neutralizer (Letheen Broth, BD: Oxford, UK) for 5 min ± 10 s. Duplicate 1 mL volumes were transferred directly from the neutralizer and from 10-fold dilutions made into diluent, to a sterile petri dish. Twenty microliters melted TSA cooled to 45 °C was added to each petri dish. Plates were incubated at 37 °C and counted at 24 and 48 h with the higher number used for biocide evaluation.

### 3.4. Statistical Analysis

An analysis of variance (ANOVA) was performed (GraphPad Prism 5.03, GraphPad Software Inc.: La Jolla, CA, USA), followed by Dunnett’s multiple comparison test against a chlorine level of 0.0 mg/L with a P value of <0.05 being regarded as significant in order to determine at what time point the free chlorine levels within the stored ECAS became not statistically different from zero.

## 4. Conclusions

This study represents the longest evaluation of the physiochemical parameters and bactericidal efficacy of ECAS within the published literature and reveals that acidic ECAS retains useful bactericidal activity for significantly longer than has been previously demonstrated and may have applications beyond those previously considered. The potent antimicrobial activity of ECAS at the point of production is widely reported in the literature, as are its credentials as a “green biocide”. Our study demonstrates that ECAS may be suited to situations where generation at the point of use is not possible due to cost or resource limitations. Furthermore, we have shown that ECAS is bactericidal in the absence of measurable chlorine. This furthers our understanding of the mechanism of action of these solutions, strongly suggesting that there are multiple biocidal components working in combination [43] rather than the antimicrobial activity being exclusively due to the presence of HOCl. Now that it has been demonstrated that ECAS can retain basic bactericidal activity for over 12 months in storage, it would be of benefit to further investigate how extended storage of this type affects the kill kinetics and antimicrobial spectrum compared to ECAS used at the point of production.
